# A machine learning methodology for the selection and classification of spontaneous spinal cord dorsum potentials allows disclosure of structured (non-random) changes in neuronal connectivity induced by nociceptive stimulation

**DOI:** 10.3389/fninf.2015.00021

**Published:** 2015-08-26

**Authors:** Mario Martin, Enrique Contreras-Hernández, Javier Béjar, Gennaro Esposito, Diógenes Chávez, Silvio Glusman, Ulises Cortés, Pablo Rudomin

**Affiliations:** ^1^Department of Computer Science, Universitat Politècnica de Catalunya. BarcelonaTechCatalonia, Spain; ^2^Department of Physiology, Biophysics and Neurosciences, Center for Research and Advanced Studies, National Polytechnic InstituteMexico City, Mexico; ^3^Barcelona Supercomputing CenterCatalonia, Spain; ^4^El Colegio NacionalMexico City, Mexico

**Keywords:** machine learning, neural signal processing, sorting of spontaneous cord dorsum potentials, capsaicin, spinal cord

## Abstract

Previous studies aimed to disclose the functional organization of the neuronal networks involved in the generation of the spontaneous cord dorsum potentials (CDPs) generated in the lumbosacral spinal segments used predetermined templates to select specific classes of spontaneous CDPs. Since this procedure was time consuming and required continuous supervision, it was limited to the analysis of two specific types of CDPs (negative CDPs and negative positive CDPs), thus excluding potentials that may reflect activation of other neuronal networks of presumed functional relevance. We now present a novel procedure based in machine learning that allows the efficient and unbiased selection of a variety of spontaneous CDPs with different shapes and amplitudes. The reliability and performance of the present method is evaluated by analyzing the effects on the probabilities of generation of different classes of spontaneous CDPs induced by the intradermic injection of small amounts of capsaicin in the anesthetized cat, a procedure known to induce a state of central sensitization leading to allodynia and hyperalgesia. The results obtained with the selection method presently described allowed detection of spontaneous CDPs with specific shapes and amplitudes that are assumed to represent the activation of functionally coupled sets of dorsal horn neurones that acquire different, structured configurations in response to nociceptive stimuli. These changes are considered as responses tending to adequate transmission of sensory information to specific functional requirements as part of homeostatic adjustments.

## 1. Introduction

It is well established that prolonged nociceptive stimulation may lead to a state of central sensitization and to the development of allodynia and secondary hyperalgesia (Traub, 1997; Arendt-Nielsen, 2015). Quite interestingly, a recent behavioral study in the mice has shown that mechanical hyperalgesia can be rendered labile and reversible after reactivation of spinal pathways during the application of protein synthesis inhibitors (Bonnin and De Koninck, 2014), or during high-dose of opioid administration (Drdla-Schutting et al., 2012), suggesting a process analogous to memory reconsolidation. Although these findings have potentially important clinical applications in the control of hyperalgesia, it must be emphasized that we know very little on the changes in the functional connectivity between the dorsal horn neuronal ensembles ensued during the state of central sensitization produced by nociceptive stimulation (see Biella et al., 1997; García et al., 2004) and of possible changes in memory reconsolidation.

Quite recently we examined in the anesthetized cat the changes in functional connectivity of dorsal horn neurons during the state of central sensitization produced by the acute section of a cutaneous nerve (Chávez et al., 2012) or by the intradermic injection of capsaicin (Rudomin et al., 2012; Contreras-Hernández et al., 2013). In these studies the strength of the functional connectivity between different populations of dorsal horn neurons was inferred from the magnitude of the correlation between the spontaneous cord dorsum potentials (CDPs) simultaneously recorded from different spinal segments. Particular attention was given to the generation of spontaneous negative-positive CDPs (npCDPs) because, in contrast with the purely negative CDPs (nCDPs), they appeared related to the preferential activation of the pathways leading to primary afferent depolarization and presynaptic inhibition (Rudomin et al., 1987, 1990; Chávez et al., 2012). Presynaptic inhibition has been considered as a key central mechanism that regulates the synaptic effectiveness of the sensory afferents in the spinal cord during normal and during pathological processes (Alvarez, 1998).

Recent studies aimed to disclose the neuronal populations involved in the generation of the spontaneous npCDPs and nCDPs were based on potentials selected from raw records using predetermined templates (Chávez et al., 2012; Contreras-Hernández et al., 2015). This procedure required continuous supervision and validation by visual inspection to extract only spontaneous nCDPs and npCDPs, which represent a small part of a wider repertory of spontaneous CDPs whose functional role remains to be established. The selection and classification of the CDPs achieved with these methods was time consuming. Therefore, we aimed to develop a procedure that would allow a faster unbiased selection and classification of spontaneous CDPs of different shapes and amplitudes. To this end we have developed a method based on machine learning to select and classify the spontaneous CDPs. We illustrate the usefulness of this method by examining the changes in spontaneous CDPs induced by the intradermic injection of capsaicin, a procedure known to produce an increased and prolonged state of central excitation (Contreras-Hernández et al., 2013).

The results obtained with the selection method presently described allowed detection of spontaneous CDPs with specific shapes and amplitudes that are assumed to represent the activation of functionally coupled sets of dorsal horn neurons that may acquire different, structured configurations in response to nociceptive stimuli. The functional implications of these findings are briefly discussed and will be examined with more detail in a forthcoming publication.

### 1.1. Plan of the work

This paper addresses our research in the following way: in Section 2 a detailed description of the data, experimental methods and general procedures is given. In Section 3 our new CDP identification methodology is fully introduced. In Section 4 we address the issue of clustering selected CDPs available from data obtained in previous experiments, while in Sections 5.1 and 5.2 we discuss two different validation procedures for the proposed methodology. Finally, in Section 6 we give our conclusions from the study and point out future work in the implementation of analytic methods for the analysis of the spontaneous cord dorsum potentials recorded under different experimental conditions.

## 2. Materials and methods

The goal of this work was to develop an automatic unsupervised classification method to achieve faster and more detailed selection and classification of spontaneous CDPs of different shapes and amplitudes recorded simultaneously from several spinal segments under specific experimental conditions. The method was devised to supersede the experts' criteria and retrieve in addition to nCDPs and npCDPs, potentially interesting CDPs that remained unselected because of the use of predetermined templates.

### 2.1. The data

In this work we used data coming from already published studies (Chávez et al., 2012; Rudomin et al., 2012) performed in anesthetized cats paralyzed and maintained under artificial ventilation (see Chávez et al., 2012 for a general description of the experimental procedures). Briefly, spontaneous cord dorsum potentials (CDPs) were recorded by means of silver ball electrodes placed on the cord dorsum in both sides of the *L*4–*L*7 spinal segments against an indifferent electrode inserted on the paravertebral muscles using AC ampliers with filters set from 0.3 Hz to 1 kHz.

**Figure 1:**
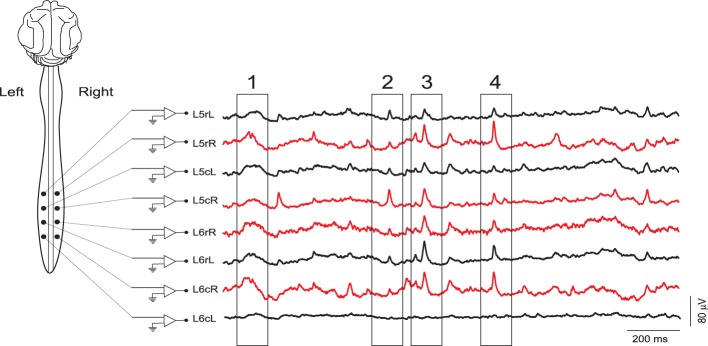
**Examples of continuous records of spontaneous CDPs from different lumbar segments in the left (black) and right (red) sides of the spinal cord during a control period**. The four boxes indicate CDPs synchronized along different lumbar segments. Some of them clearly defined as nCDPs (box 2, L5cR; box 4, L6rL) or npCDPs (box 3, L6rL; box 4, L5rR). L, left; R, right; c, caudal; r, rostral. Data obtained from experiment *060911* (negative voltages plotted upward).

In the experiment *300103* the CDPs were recorded under deep anesthesia without additional maneuvers. In the experiment *060911* the CDPs were recorded for several minutes during a control period (see Figure 1) and also at different time intervals after the intradermic injection of capsaicin in the plantar surface of the left footpad (30 μ*l* of 1% solution).

### 2.2. Visual identification of CDPs

The method used in previous studies to sort the spontaneous CDPs according to their shapes and amplitudes (Chávez et al., 2012), was based, first, on performing a small sample visual selection (order of one hundred) of nCDPs and npCDPs. Then, by using their respective averages as fixed templates to retrieve the nCDPs and npCDPs from the whole sample. Usually three experts inspected the preselected CDPs, leaving those potentials that were clearly nCDPs or npCDPs. The selection of nCDPs and npCDPs usually took several hours and made necessary the design of a faster and reliable procedure to retrieve and classify the different types of spontaneous CDPs. This procedure, based on template matching and prior expert knowledge to search for typical CDPs shapes, should be considered as a supervised detection method. It implicitly considers a basic set or dictionary of possible CDPs made of only two recurrent classes of spontaneous CDPs (nCDP and npCDP) learned by the experts from their experience.

### 2.3. Phases involved in CDP identification

The spontaneous spinal activity (SSA) registered from the cord dorsum in a given spinal segment can be seen as a multivariate time series. This series can be divided into a *control period*, lasting typically 10–20 min, followed by similar recording periods after the intradermic injection of capsaicin. With a sampling frequency between (*f*_*s*_ ~ 5 − 10 kHz), and the recording times presently used the experiments result in a multivariate time series composed by several million points. The description of the identification phases is as follows (see Section 2.5 for a summary):

The *first phase* of the analysis consists in extracting the CDPs from the recorded data as subsequences of a time series. For this task, we define a time series as an ordered set of observations of length *T*_*N*_. A subsequence *S*_*n*_ is a sampling subset of length *T*_*n*_ ≪ *T*_*N*_ of continuous positions *S*_*n*_ = {*t*_*p*_, …, *t*_*p*+*n*_} for 1 ≤ *p* ≤ *N* − *n* + 1. Subsequences from a time series can be collected using a sliding window of fixed size (*T*_*w*_) moving across the data. A possible way of identifying CDPs from the subsequences is to determine if a subsequence *S*_*n*_ is similar to other subsequences in the time series. We would consider in this case that subsequences form recurring patterns (nCDPs or npCDPs or others). In order to define if a subsequence belongs to a similar pattern (i.e., similar shape) it is necessary to satisfy certain minimal constraints: subsequences must have a similar behavior in terms of temporal variation, the similarity between pairs of subsequences must be higher than a given threshold and, finally, that subsequences should not overlap each other.

The *second phase* involves the extraction of features for a better characterization of the CDPs, performed either automatically or defined by the user. Quite often, it is advisable to apply some feature extraction approach to raw signals before an automatic classification procedure. In order to capture the possible shapes, it is necessary to use as features not only amplitude and duration, but also an initial baseline, which may be interpreted as a steady state condition for the signal.

At this stage, noise reduction and feature selection also have to be addressed. Principal Component Analysis (PCA) (Jolliffe, 2002) can be used to get a linear decomposition of the data capturing its maximal variance. PCA basically finds an ordered set of orthogonal basis vectors providing the directions in the data with the largest variation. The principal component vectors are obtained by computing the eigenvectors of the covariance matrix of the data. Moreover, ordering the eigenvalues and using some threshold might also be used as a dimensionality reduction technique.

The *third phase* involves the identification of recurring classes using cluster analysis (Duda et al., 2000) which can be used for automatically finding clusters in multidimensional data sets. A basic assumption underlying clustering methods is that the data are generated from several independent classes, each of which can be described by a relatively simple model. Hereafter by classes we mean amplitudes and shapes of recurring CDPs candidates close to a given cluster using some similarity measure.

Using cluster analysis we may describe the cluster prototype, the variability of the data around these prototypes and the characteristics of the clusters. Cluster analysis in general makes no assumptions about CDPs shapes which might be considered suboptimal. To mimic the expert behavior we assume that CDPs shapes are smooth, which helps finding the relevant information, leaving to the cluster analysis the task to identify recurring patterns.

In addition to the identification and selection of spontaneous CDPs with specific shapes and amplitudes, the method should allow detection of specific combinations of CDPs simultaneously generated in different segments (see boxes in Figure 1). A possible approach would be to count, for each combination of basic patterns, the number of occurrences. Then one could use this count to define the *configuration* of functional interconnections between the neuronal populations involved in the generation of the CDPs in different spinal segments, giving detailed information on the temporal recurrence relations between identified patterns. This has the potential to identify functionally defined changes in the internal connectivity of the neuronal ensembles that generate the spontaneous CDPs during the performance of different tasks (Manjarrez et al., 2000; Contreras-Hernández et al., 2015).

### 2.4. Related work

The problem addressed by the first phase of the methodology is similar to the analysis of neural spike activity made with Spike Sorting Methodology (SSM) (Lewicki, 1998). Nevertheless, we have to take into account that CDPs have amplitudes between 5 and 250 μV and durations between 20 and 300 ms, because these CDPs result from the activation of either the same or different neuronal ensembles (Contreras-Hernández et al., 2015). Henceforth, the possible patterns of these activations are quite different from those studied using SSM, meaning that a more general perspective has to be used to analyze such kind of signals.

The problem of efficiently locating patterns in a time series can be addressed in terms of knowledge discovery through the automatic detection of frequently occurring patterns. In principle, this task can be accomplished by applying Frequent Motif Discovery (FMD) methods for time series (Lin et al., 2002). Within this approach, given a window size, a pattern is extracted if it repeats with a frequency higher than a predetermined value. Although we are interested in recurring patterns, we have not considered their occurrence frequency. Furthermore, the methods used in FMD resort to approximated algorithms due to the computational complexity of the problem, and we are interested on identifying all the occurring patterns.

Related to the second phase, in spike sorting, feature analysis is used to identify spikes having similar duration or amplitude within some given boundaries. However, choosing features based on intuitive ideas, while simple in principle, can often yield to poor pattern separation.

The third phase approach is also common to potential sorting, but in our case, the patterns are selected using as features the amplitude and duration of the peaks above a given threshold defined by the user, aiming to include *most* of potentially eligible candidates. In potential sorting, the cluster analysis usually yields a limited number of (1–4) independent classes and it is relatively easy to identify the number of classes. CDPs selection could be also performed using a similar methodology. However, being the peaks the result of activation of neuronal populations of different magnitudes, the complexity and number of independent shapes should be expected to be much larger and more difficult to assess.

### 2.5. Our approach to automated identification

Considering all these concepts together we devised an semi-automatic classification method able to identify CDPs from the raw data recordings of each spinal segment comprised of five different phases:

1. **CDPs candidate detection**: during this phase raw CDPs recorded from given segment are analyzed using a sliding window to obtain subsequences from the time series. They are then selected according to some given constraints and smoothness that mimics expert's knowledge used to identify the different classes of CDPs.
2. **CDPs feature extraction**: during this phase the data are preprocessed in order to maximize the information related to the CDPs shapes, reducing noise and data dimensionality.
3. **CDPs cluster analysis**: during this phase the candidate CDPs are analyzed using PCA (Jolliffe, 2002) as feature selection method and then, using the *k*-means clustering algorithm (Duda et al., 2000) a basic set or dictionary of recurring independent classes is built (shapes dictionary). Patterns of the identified classes define the recurring shapes of CDPs for each spinal segment.
4. **CDPs occurrence analysis**: during this phase we study correlation and synchronization between different time series by counting the number of occurrences and concurrences of the synchronized CDPs classes according to a given temporal resolution. The goal of this phase is to identify possible relations among concurrent CDPs.
5. **CDPs recurrence analysis**: during this last phase we search for temporal recurrence relations between concurrence of identified patterns of synchronized classes of CDPs. This may disclose possible temporal relations among consecutive groups of CDPs.

In this paper, we focus on the first three steps as we will address the last two in a future publication.

## 3. CDP candidate detection

We are interested in building an automated and unsupervised CDPs detection method using some smoothness in the definition of the CDPs candidates. The chances of detecting a signal embedded in noise are improved when one can take advantage of prior information about the signal and the noise. The prior information could be acquired through experimental trials. Hence, to keep the algorithm general and unsupervised, the prior information must be loose. We have assumed that the noise contained in SSA background is stationary, essentially Gaussian and also independent from the neuronal signal (Solodkin et al., 1991). Although these assumptions are not crucial for our implementation, they ensure the mathematical tractability of the derivations.

The problem of detecting *transients* (meaning a significative and structured variation of the signal with respect the background noise) in a collection of noisy observations has been extensively studied. The presence of a useful signal in a background noise casts as a hypothesis testing problem, where no signal is present under the null hypothesis. In case of unsupervised problems, the signal to be detected is not well known, so no uniformly powerful test can be used. As a result, the detector performance relies on the signal representation. A common practice is to use a model based or expansion based signal representation. In the absence of a signal model, one may project onto a canonical set of basis functions, working then with the set of expansion coefficients. Depending on the signal representation, the detection problem can be formulated in time domain, frequency domain or time-frequency domain. Hereafter, we use frequency domain through the Discrete Fourier Transform (DFT) expansion. CDPs candidates may be identified as subsequences in a time series of duration *T*_*N*_ using a sliding window of duration *T*_*w*_ ≪ *T*_*N*_. As a constraint, we set the sliding window to move across the time series with a resolution of *T*_*r*_ to avoid possible overlaps. The finite set of basis frequencies is then determined by the sampling rate of the signal *f*_*s*_ = *O*(*kHz*) and its duration *T*_*w*_ with *N*_*w*_ = *T*_*w*_*f*_*s*_ the number of samples of the discrete signal. Expert knowledge is used to restrict the frequency range of the CDPs signal with respect to possible SSA noise background.

Methods used to identify transients in presence of noisy background comprise, for example, a window discriminator detecting signals exceeding a simple amplitude threshold and passing through user-specified time-voltage boxes. This method requires human supervision, its manual nature makes it unreliable and its statistical properties are not well understood. Another possibility is represented by the amplitude discrimination where the threshold value can be set automatically. Performance of this method may deteriorate rapidly under low Signal-to-Noise Ratio (SNR) conditions. Matched filters, which are known to maximize SNR when a signal is embedded in a white noise, can also be used. However, this method requires building templates and is not unsupervised. Finally, power detection may be used to evaluate the instantaneous power of the signal using a sliding window, which is compared against a threshold derived from the mean and the standard deviation of the noise power. Performance of this method is known to be poor in a low SNR environment.

The CDPs candidate detection can be formulated as a binary hypothesis testing problem where under the null hypothesis H_0_ the signal is not present and under the alternative hypothesis H_1_ both signal and noise are present:

(1) $$\begin{array}{r} H_{0}\;:\;x\left[t\right]=n\left[t\right]\;t=1,\ldots ,N_{w} \end{array}$$

(2) $$\begin{array}{r} H_{1}\;:\;x\left[t\right]=s\left[t\right]+n\left[t\right]\;t=1,\ldots ,N_{w} \end{array}$$

**Figure 2:**
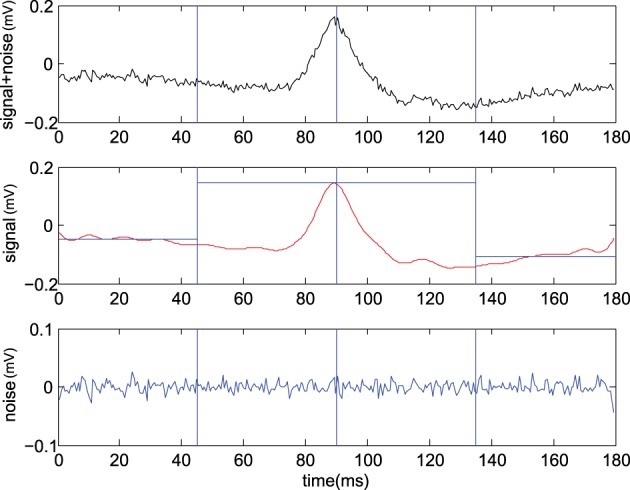
**Example of CDP candidate selection**. **(Upper)** typical raw signal $ŝ\left[t\right]+{\hat{n}}_{0}\left[t\right]$, **(Center)** identified signal using IDFT with center $\hat{x}=IDFT\left({\tilde{X}}_{s}\right)$ where we also report left ${\hat{x}}_{l}$ and right ${\hat{x}}_{r}$ average levels (see text), **(Lower)** remaining Gaussian noise ${\hat{n}}_{s}=IDFT\left({\tilde{X}}_{n}\right)$ (From experiment *300103*).

*x*[*t*] represents a noisy observation (evidence) at a discrete time *t*, *s*[*t*] is the transient (the CDP candidate) to be detected and *n*[*t*] the background noise. Figure 2 shows one example of the CDP signal in presence of Gaussian noise. Moreover, multiple transients could be present representing the main differences between the problems of the classical signal detection and the detection of CDPs candidates. By linearity of the DFT, being $\tilde{X}=DFT\left(x\right)$, the two hypothesis can be reformulated as

(3) $$\begin{array}{r} H_{0}\;:\;\tilde{X}\left[k\right]=\tilde{X}\left[k\right]\;k=1,\ldots ,N_{w} \end{array}$$

(4) $$\begin{array}{r} H_{1}\;:\;\tilde{X}\left[k\right]=\tilde{S}\left[k\right]+\tilde{X}\left[k\right]\;k=1,\ldots ,N_{w} \end{array}$$

As in any hypothesis testing problem, the goal is to determine whether the evidence supports the rejection of H_0_. This decision should be made optimally with respect to a suitably chosen objective function. Let $P\left(H_{0}|\tilde{X}\right)$ and $P\left(H_{1}|\tilde{X}\right)$ be conditional probabilities associated with accepting and rejecting the hypothesis H_0_ given the evidence $\tilde{X}$, respectively. The optimal decision rule minimizing the overall cost is to accept the hypothesis with a smaller conditional probability according to

(5) $$\begin{array}{r} P\left(H_{0}|\tilde{X}\right){≶}_{H_{1}}^{H_{0}}P\left(H_{1}|\tilde{X}\right) \end{array}$$

which using the Bayes theorem $P\left(H_{i}|\tilde{X}\right)=p\left(\tilde{X}|H_{i}\right)P\left(H_{i}\right)∕p\left(\tilde{X}\right)$ becomes

(6) $$\begin{array}{r} \frac{p\left(\tilde{X}|H_{1}\right)}{p\left(\tilde{X}|H_{0}\right)}{≶}_{H_{1}}^{H_{0}}\gamma \end{array}$$

with an user defined acceptance threshold typically γ ≫ 1.

**Figure 3:**
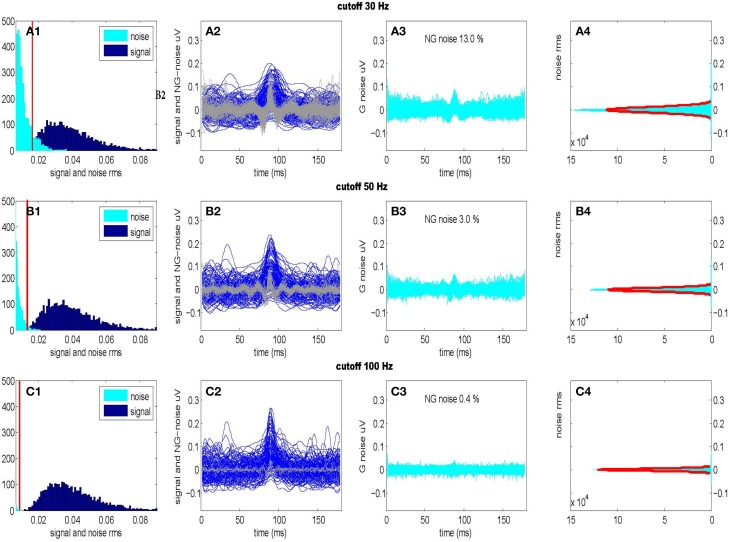
**Optimal separation of signal and noise for three different frequency intervals**. (Upper): *f*_*B*1_ = [0–30] Hz; (Center): *f*_*B*2_ = [0–50] Hz; (Lower): *f*_*B*3_ = [0–100] Hz. Firsts panels show signal and noise (RMS) histograms **(A1,B1,C1)** separated using respective frequency intervals *f*_*B*1_, *f*_*B*2_, *f*_*B*3_ following the procedure described in Figure 2. Considering the distribution of the noise, we also show the RMS threshold separating non-Gaussian from Gaussian noise. Second panels **(A2,B2,C2)** show the time windows of the signals (darker) and of the non-Gaussian (NG) residual noises (lighter) presented in the previous histograms. Third panels **(A3,B3,C3)** show the time windows of the residual Gaussian (G) noises as well as the percentage of residual non-Gaussian noise still present. Last panels **(A4,B4,C4)** show the amplitude histograms (with a Gaussian fit) for the residual noises **(A3,B3,C3)** (Data taken from segment L6cL, from experiment *300103*).

For purposes of unsupervised signal detection, we must separate $\tilde{X}$ by estimating the noise level at each frequency from the sampled data. We can obtain these estimates by accepting only frequencies in a given band *f*_*B*_ followed by the inverse DFT transform. In our case, the frequency band *f*_*B*_ becomes part of the hypothesis testing procedure at the level of coefficients. In practice with CDPs of duration something between 100 and 300 ms we may filter out the CDPs signal in the frequency band *f*_*B*_ ⊂ [0, 100] Hz while the components outside this range can be treated as gaussian stationary noise. Such a procedure allows to separate $\tilde{X}$ in two disjoint contributions depending on the frequency interval, a signal component ${\tilde{X}}_{s}$ in case {*f* ∈ *f*_*B*_}, and noise component ${\tilde{X}}_{n}$ when {*f* ∉ *f*_*B*_}. Accordingly, we may separate signal from the noise in the time domain evaluating the Inverse DFT as $\hat{x}=IDFT\left({\tilde{X}}_{s}\right)$ under the hypothesis H_1_. This assumption works fine in practice as we need, to some extent, a loose way of detecting transients candidates. In Figure 3 we show the RMS of signal and noise, separated using three different frequency intervals (*f*_*B*1_ = [0, 30] Hz, *f*_*B*2_ = [0, 50] Hz, *f*_*B*3_ = [0, 100] Hz) and the effect on the normality of the residual noise. We optimize the frequency interval *f*_*B*2_ = [0, 50] Hz accepting a small deviation from gaussianity (~3%) which is mainly due to the non separable signal component still present into the residual noise.

Using the linearity of the IDFT we may reformulate

(7) $$\begin{array}{r} H_{0}\;:\;\hat{x}\left[t\right]={\hat{n}}_{0}\left[t\right]\;t=1,\ldots ,N_{w} \end{array}$$

(8) $$\begin{array}{r} H_{1}\;:\;\hat{x}\left[t\right]=ŝ\left[t\right]+{\hat{n}}_{0}\left[t\right]\;t=1,\ldots ,N_{w} \end{array}$$

where ${\hat{n}}_{0}\left[t\right]\ll \hat{n}\left[t\right]$ is the residual noise. Hence the decision rule can be safely applied at the maximum value of the signal ${\hat{x}}_{m}=\max\left(\hat{x}\right)$, We report an example of the CDP signal to noise separation procedure in Figure 2. Assuming the residual noise to be Gaussian one may write $p\left({\hat{x}}_{m}|H_{0}\right)\sim N\left(0,\sigma_{0}^{2}\right)$ and $p\left({\hat{x}}_{m}|H_{1}\right)\sim N\left(\mu_{m},\sigma_{0}^{2}\right)$ and the decision rule becomes

(9) $$\begin{array}{r} |{\hat{x}}_{m}|{≶}_{H_{1}}^{H_{0}}\;\frac{\mu_{m}}{2}+\frac{\sigma_{0}^{2}}{\mu_{m}}\log\left(\gamma \right) \end{array}$$

where μ_*m*_ is the mean value of $\left|{\hat{x}}_{m}\right|$ under the hypothesis H_1_ and σ_0_ is the standard deviation of ${\hat{x}}_{m}$. The parameters (μ_*m*_, σ_0_, γ) are not known and have to be estimated from the data. In practice we set a threshold on the maximum of the filtered signal ${\hat{x}}_{m}>x_{thd}=0.05$ μV determined experimentally.

### 3.1. Noise estimation

While this test only verifies the presence of transients, we also examined the quality of the transients. For example, in a time series with *control period* lasting 1200 s with sampling frequency *f*_*s*_ = 1670 Hz, a time window of *T*_*w*_ = 180 ms and a time resolution of *T*_*r*_ = *T*_*w*_ / 12 = 15 ms, the algorithm checks 60,000 time windows detecting on average 4000 transients. A fast quality cut is used to check badly defined CDP candidates, according to the prior assumption of smoothness. Intuitively this can be accomplished by measuring the ratio between the CDP candidates maximum amplitude and the left and right sides average within a given tolerance. By dividing the time window *T*_*w*_ in four parts of equal size *T*_*p*_ = *T*_*w*_ / 4 we have the CDP left ${\hat{x}}_{l}$ and right average ${\hat{x}}_{r}$, averaging the signal in the time interval [0, *T*_*p*_] and [*T*_*w*_ − *T*_*p*_, *T*_*w*_], respectively. The quality cut selects only those CDP candidates having ${\hat{x}}_{m}>p_{smth}{\hat{x}}_{r}$ and ${\hat{x}}_{m}>p_{smth}{\hat{x}}_{l}$ (we used *p*_*smth*_ = 1.5 determined experimentally). On average, this quality cut has an efficiency of around 80%. Accordingly, using the previous example, this leads to 3200 transients surviving as CDP candidates.

**Figure 4:**
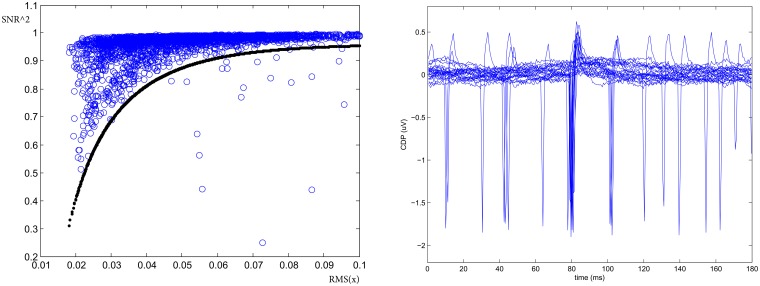
**SNR coherence test**. **(Left)** *SNR*^2^ vs. RMS allows for the identification of large deviation from gaussianity into the noise. **(Right)** One example of spurious potentials recorded on the cord dorsum in left segment L4 identified with the test (From experiment *300103*).

Finally, the presence of large deviation from gaussianity in the noise might be detected using a SNR coherence test. Defining the Signal-to-Noise-Ratio as:

$$\begin{array}{l} Y_{snr}=Mean\left[{\left(\frac{s}{s+n}\right)}^{2}\right]≃Mean\left[{\left(\frac{\hat{x}}{x}\right)}^{2}\right]=|{\tilde{X}}_{s}{|}^{2}∕|\tilde{X}{|}^{2} \end{array}$$

and looking at its functional with respect to the standard deviation *X*_*rms*_ = *RMS*(*s*+*n*) = *RMS*(*x*), it allows for the identification of large non-Gaussian noise deviation. This is shown on the left panel of Figure 4 where the curve defines the acceptance region of normal (Gaussian noise) behavior for a given sensor defined as $Y_{snr}^{thd}=p_{snr}\left(1\right)\frac{1-p_{snr}\left(2\right)e^{-p_{snr}\left(3\right)X_{rms}}}{1-p_{snr}\left(4\right)e^{-p_{snr}\left(3\right)X_{rms}}}$ where the parameters *p*_*snr*_ = (0.96, 2, 1, 50) are determined experimentally. Hence to detect the presence of large deviation from gaussian noise background in the time window we check if $Y_{snr}<Y_{snr}^{thd}$. On average this quality cut has an efficiency ranging between 90 and 100% depending on the behavior of the segment. The right panel of Figure 4 shows one example of detected occasional transients of unknown external sources and correctly identified by this procedure as large deviation from gaussianity into the noise.

### 3.2. Performance evaluation

For the evaluation of the performance we may consider the CDPs identification as the retrieval of relevant information treated as a two-class problem. A classifier usually labels examples as either *Positive* or *Negative* and the decision made by the classifier can be represented in a confusion matrix having four categories: *False Positives, True Positives, False Negatives*, and *True Negatives*. Performance of the CDPs detection phase can be evaluated by looking at the *Precision* (fraction of retrieved instances that are relevant) and the *Recall* (fraction of relevant instances that are retrieved) (Davis and Goadrich, 2006).

Precision and Recall for the CDPs candidate detection phase have been estimated using the Control period for the experiment *060911*, for the L4 to the L7 lumbar segments, and are reported in Table 1. The Recall and Precision are proportions, thus the according confidence intervals can be calculated by using standard statistical methods for difference between proportions (Newcombe, 1998). We used the experts' knowledge in order to discriminate the relevant transients in the selected period as well as the retrieved transients which were not selected by the detection algorithm. The data depicted in Table 1 show that the estimates of Recall and Precision fractions for the CDPs selected from different spinal segments (e.g., L4cL, caudal part (c) of the left (L) L4 segment, L5rR, rostral part of the right (R) L5 segment, etc.) varied between 0.74–0.81 and 0.83–0.87, respectively.

**Table 1:** **Mean and standard deviation of Recall and Precision for the CDPs candidate detection phase evaluated using the control period for the experiment *060911***.

	**L4cL**	**L4cR**	**L5rR**	**L5rL**	**L5cR**	**L5cL**
Recall	0.79± 0.04	0.78± 0.06	0.77± 0.06	0.80± 0.04	0.74± 0.04	0.81± 0.03
Precision	0.85± 0.04	0.84± 0.05	0.86± 0.05	0.81± 0.03	0.87± 0.03	0.85± 0.03
	**L6rR**	**L6rL**	**L6cR**	**L6cL**	**L7rL**	
Recall	0.79± 0.03	0.79± 0.03	0.78± 0.03	0.79± 0.03	0.77± 0.04	
Precision	0.85± 0.03	0.85± 0.03	0.85± 0.03	0.84± 0.03	0.83± 0.04	

Further explanations in text.

We considered that on average, the detection phase algorithm shows a relatively good quality in terms of these indicators, since the changes in the probability of occurrence of different classes of CDPs (see Section 5.2) are consistent with previous observations based on correlation measurements that indicated stability in the functional connectivity among different lumbar segments during the control period (Rudomin et al., 2012).

**Figure 5:**
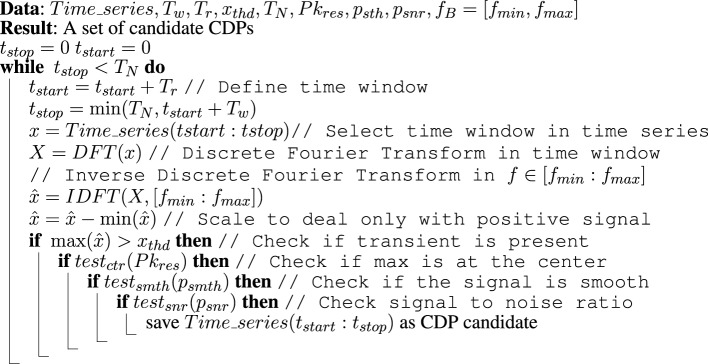
**CDPs candidate selection algorithm**.

Figure 5 displays the steps required to select a specific time window as CDP candidate. The algorithm requires several parameters which have to be defined looking at some performance measure. In particular, we set the time window in the interval *T*_*w*_ = [100, 300] ms as defined by the experts; *f*_*B*_ = [0, 50] Hz, the time resolution step is fixed as *T*_*r*_ = *T*_*w*_ / 12, the threshold on the max of the signal to detect a transient *x*_*thd*_ = 5 μ*V*, the resolution on the position on the max of the peak *Pk*_*res*_ = *T*_*w*_ / 12 the smoothness parameters *p*_*smth*_ = 1.5 and the non-Gaussian coherence test parameters *p*_*snr*_ = (0.96, 2, 1, 50).

Validation of the method and optimization of the parameters used in the present selection method has been performed using a set of nCDPs and npCDPs selected by experts from the raw data and performing the experiment described in Sections 5.1 and 5.2. This selection method was able to correctly identify all the events marked as interesting (nCDPs or npCDPs) by the experts.

## 4. Clustering of selected CDPs

After CDPs candidates have been identified using the selection procedure described, the steps before the generation of the basic set or dictionary of CDPs proceed as follows. The time-stamp of a detected CDP corresponds to the time of its maximum value in the selected time window. Using the expert, we define a suitable time window around the identified transient maximum. So, for instance, the expert can decide using a sampling frequency *f*_*s*_ = 1670 Hz and a duration of *T*_*w*_ = 180 ms resulting in a representation of a CDP sampled in 300 points.

After selecting the time window, data is preprocessed in order to prepare it for clustering. Selected CDPs in time windows of duration *T*_*w*_ are smoothed using a low pass filter (*f*≤ 50 Hz). To ease the comparison of different CDPs a potential offset is removed by subtracting the average of the first 100 and last 100 points of the signal. Given that the signal must be sufficiently smooth, CDPs are processed by using PCA features extraction to compute the 10 most relevant dimensions that describe the whole set of identified CDPs and using only those dimensions to reconstruct each CDP.

We tried a number of clustering approaches such as *k*-means, *k*-medoids, EM Gaussian Mixtures, DBSCAN, Affinity-propagation (Gan et al., 2007), and we selected the well known *k*-means algorithm because resulting cluster prototypes were interpretable and meaningful for the experts. Also, the obtained clusters divided the nCDPs and npCDPs almost into disjoint clusters.

For *k*-means the main issue is to select the number of clusters. Given that there is not *a priori* information about the number of different classes, the decision has to be related to cluster quality. Since this algorithm is also dependent on initialization, we will use this circumstance to find the adequate number of clusters. We will consider that the *correct* number of clusters corresponds to the value that yields the more *stable* results. In this context, stable means that, given a number of clusters and several initializations, the mean similarity among the clusterings of the data is larger than for other number of clusters.

There are several possible measurements that can be used as clustering similarity. Despite selecting one specific clustering algorithm, we want also to be able to compare the results obtained with other methods. This constrains the possible measurements to those that are independent of cluster representation. These kind of measurements are known as external cluster validity indexes (Halkidi et al., 2001) and they only consider how the different examples are grouped into clusters. From those measures we have selected the *Adjusted Mutual Information score* (AMI) defined in Vinh et al. (2010). This is an information theoretical measure that compares the coincidence of two partitions. It has the additional characteristic over simple mutual information of being corrected for chance. For *U* and *V*, two different partitions of a dataset, the AMI score is defined as:

(10) $$\begin{array}{r} AMI\left(U,V\right)=\frac{MI\left(U,V\right)-E\left[MI\left(U,V\right)\right]}{\max\left(H\left(U\right),H\left(V\right)\right)-E\left[MI\left(U,V\right)\right]} \end{array}$$

where *MI*(*U, V*) is the mutual information of two partitions, *E*[*MI*(*U, V*)] the expected mutual information between two partitions and *H*(*U*) the entropy of a partition. This measure is bounded in the interval [0, 1], where 1 is the maximum similarity.

**Figure 6:**
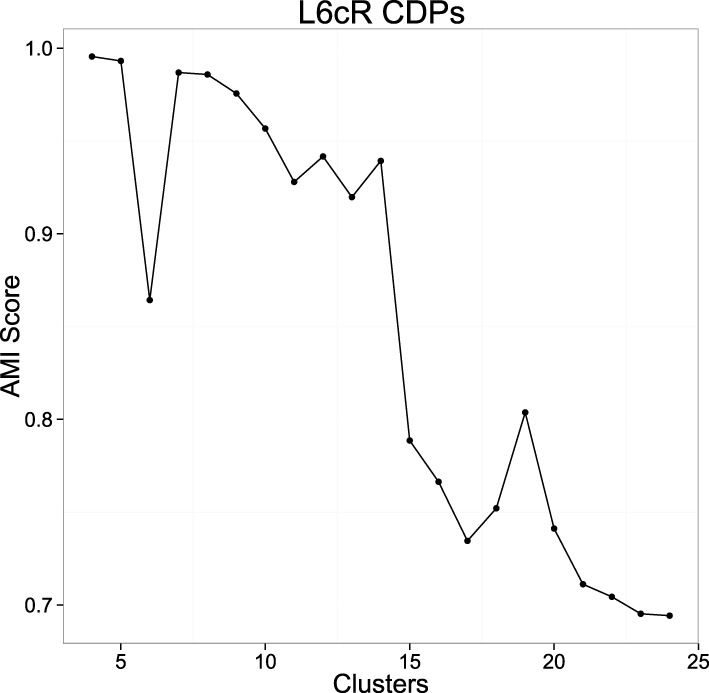
**Example of the mean AMI Score evolution with respect to the number of clusters using experiment *060911***. The picture shows the result of the stability for the clusterings using the proposed measure in a range from 4 to 25 (see text).

In order to reduce the initialization dependence of *k*-means we used the *k*-means++ (Arthur and Vassilvitskii, 2007) initialization heuristic, that has been shown to improve the results of this algorithm. This heuristic uses randomization, so it is not deterministic. This allows testing the stability of the clusterings using the proposed measure. The more adequate number of clusters will show a more compact and stable set for different initializations. For the number of clusters we choose a range between [4–25]. For each value we obtained a number of clusterings (40) with the *k*-means algorithm, each being the best of 10 random initializations using the mentioned heuristic. For each cluster number, we computed the mean AMI of all the pairs of clusters. Figure 6 shows the results using data from experiment *060911*. The AMI score is usually larger for a very small number of clusters, this value decreases as the number of clusters increases and usually presents one or more high peaks for the more stable clusterings.

Once AMI score graph is computed, the values of *k* that correspond to peaks in the picture are collected and tested in order to ensure that the classifications obtained with such *k* numbers are not spurious. So for each *k* that produces a significant peak in the picture, 10 executions of the *k*-means algorithm are done to obtain 10 dictionaries. Each execution of *k*-means is the best on 100 random initializations, so we try to obtain for each dictionary a classification close to the global minimum that *k*-means tries to find. After the 10 dictionaries are generated, they are compared in order to know if they are equivalent or not. Two dictionaries *d*_1_ and *d*_2_ are equivalent when, for each class in one dictionary, there exists another class in the other dictionary that share more than 90% (which is a parameter) of the elements that form both classes. So we are able to assign one class from one dictionary to another class on the other dictionary. This assignment of equivalent classes is made by an implementation of the Hungarian algorithm for weighted bipartite graphs matching (Lovász and Plummer, 2009). When 10 different executions of *k*-means in these terms return 10 equivalent dictionaries, we can be pretty sure that the dictionaries are not spurious and so they have statistical relevance. So, for each *k*-value corresponding to a peak in the AMI score graph, we remove those that do not produce equivalent dictionaries for different executions of *k*-means. From the set of surviving *k*-values, we select the highest ones because they can help to discover a larger number of statistically significant classes from the data and show more diversity. Once selected one or several possible candidates for the number of clusters, the groups obtained can be examined by the experts to decide the best one. After that, the resulting clustering is fixed as the dictionary for the possible CDPs shapes and used for subsequent analysis tasks.

## 5. Results

### 5.1. Experimental evaluation: mapping nCDPs and npCDPs into dictionaries

One way to test the dictionaries generated by the proposed methodology is to check in the generated dictionaries the appearance of *negative* (nCDPs) and *negative positive* (npCDPs) defined by the experts. To this end, we asked two experts who previously performed the CDPs template selections to check the raw potentials recorded in the left L6 segment in experiment *300103* to find at least 100 clear examples of nCDPs and npCDPs. These experts marked the time of occurrence of the CDP peaks and labeled them 166 CDPs as nCDPs and 26 as npCDPs. The set of selected examples was used to test the proposed methodology. Since our intention was to keep the dictionary generation procedure based using unsupervised learning mechanisms, supervisor identification was limited to the identification of clear nCDPs and npCDPs.

In parallel with the visual selection of nCDPs and npCDPs, the methodology was applied on a sequence of 500 s of raw recordings. The experiment evaluates whether or not the expert selected CDPs appear in the generated dictionary, which express the *Recall* of interesting signals into the dictionary. We also checked if nCDPs and npCDPs appeared in different classes and tested the generated dictionary ability to represent them. Applying the methodology described in Section 2, after CDPs selection, data in each time window were preprocessed using a low pass filter (*f*≤50 Hz), removing the baseline and applying PCA as feature selection. This allowed the identification of 1266 CDPs candidates during the 1200 s of the *control period*.

**Figure 7:**
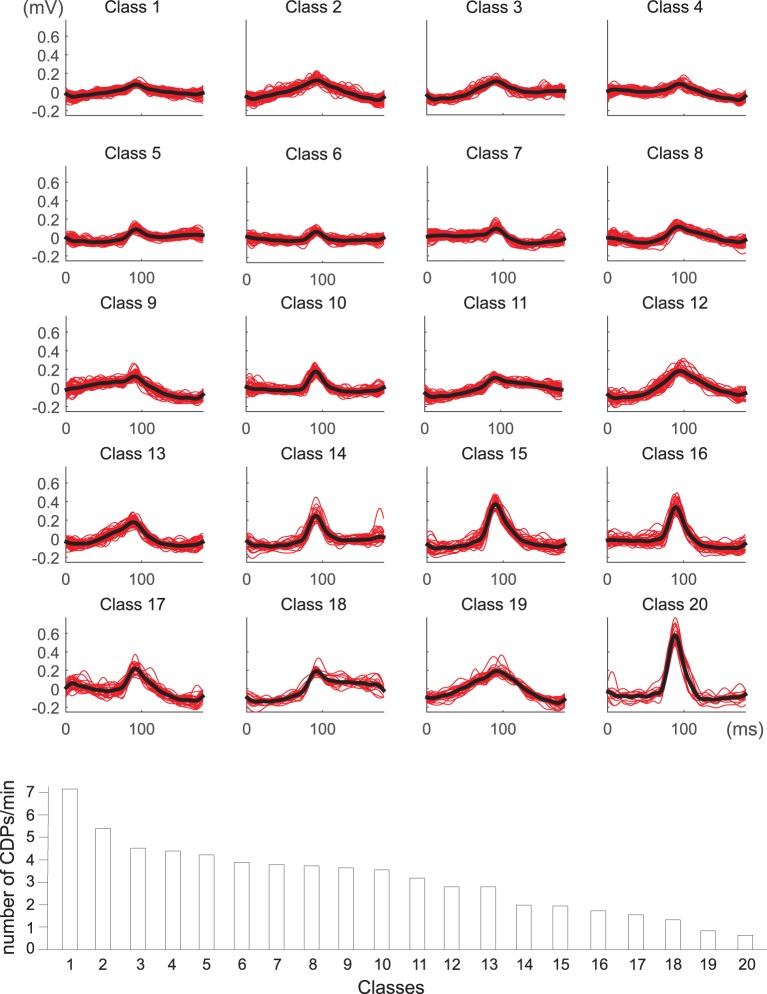
**Dictionary obtained for *k* = 20 classes of spontaneous CDPs recorded in L6 left lumbar segment in experiment *300103***. Clear npCDPs are shown in classes 7 and 16 and clear nCDPs mainly appear in classes 6, 10, 14, and 15. The lower histogram illustrates the frequency of appearance of each class during a recording time of 20 min. Note that classes with the smallest amplitude appear more frequently than largest classes of spontaneous CDPs. See details in text.

The identified CDPs were preprocessed with the *k*-means algorithm looking for the most stable classifications which were obtained with *k* = 12, 17, and 20. After examination of the different possibilities by the experts, the classification with 20 clusters was considered the most representative one (see Figure 7). An automatic tagging of classes as N, NP classes and other classes was made by finding CDPs labeled by the experts in each class. When the proportion of user-labeled nCDPs or npCDPs in a class was 10 times higher than the expected number of cases using a random assignment, the class was pre-classified as a candidate to represent that CDP. In this way, classes 6, 10, 14, and 15 were pre-labeled as candidates to represent nCDPs while classes 7 and 16 were pre-labeled as candidates to represent npCDPs. Final examination of the classes by the experts confirmed that classes 6, 10, 14, and 15 should effectively be labeled as nCDPs. Accordingly, classes 7 and 16 were considered by the experts as npCDPs. Class 17 was also considered by experts as an example of npCDPs class. This class was not discovered by the automatic method for post-tagging classes because of the small number of npCDPs used as examples (only 26 cases).

**Figure 8:**
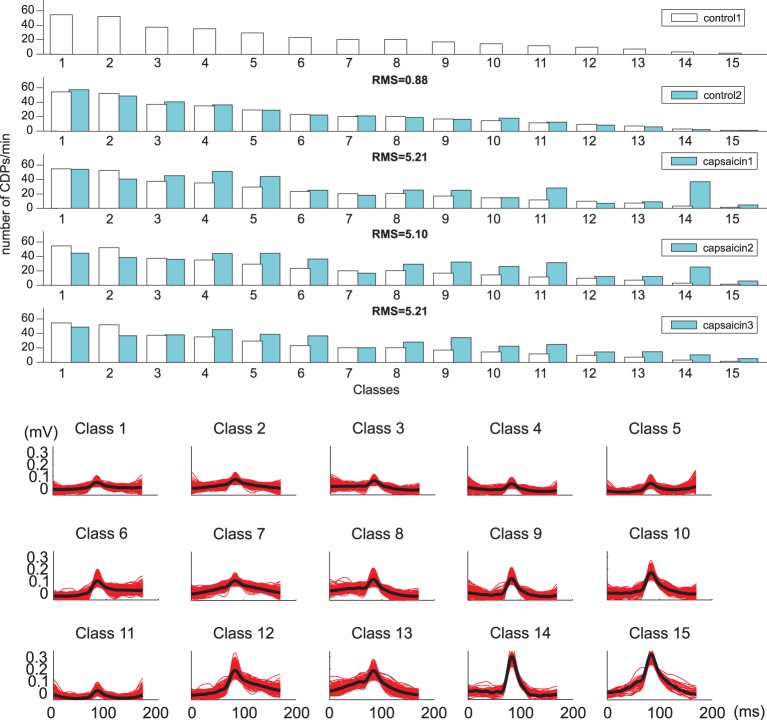
**(Upper) Classes (*k* = 15): histograms show variations in frequency of occurrence of the different classes of CDPs obtained during the control and capsaicin recording periods made in the *L6* left rostral lumbar segment in the anesthetized cat from experiment *060911* (white bars representing control1 are plotted in each histogram for quantitative comparison)**. Lower set of traces show the 15 classes of selected CDPs used tho construct the histograms displayed above.

The histogram displayed in the lower part of Figure 7 shows the frequency of the 20 different classes of CDPs retrieved from a 20 min sample period plotted in decreasing order. It may be seen that the smallest potentials (e.g., classes from 1 to 7) appeared more often than the largest potentials (e.g., classes from 14 to 20). This was a recurrent finding as illustrated by the upper pair control histograms in Figure 8 obtained from data recorded in experiment *060911*.

### 5.2. Experimental evaluation: capsaicin experiment

Once we had established the ability of the method for proper selection and classification of the different classes of spontaneous CDPs, it seemed desirable to examine the extent to which the different classes of CDPs changed at different times before and after the intradermic injection of capsaicin (3μl of 1% solution) into the left hindlimb footpad because previous experiments (Contreras-Hernández et al., 2013) have shown that the intradermic injection of capsaicin changes the patterns of functional connectivity among the dorsal horn neural networks involved in the generation of spontaneous CDPs.

For assessing the difference among the distribution of the CDPs in the different periods we defined a measure based of the Root Mean Square (RMS) difference among the histogram of their distribution (see Appendix A.1).

The fifteen classes of CDPs illustrated in the lower part of Figure 8 were retrieved from experiment *060911*, using the whole database that included recordings made during two control periods and at different times after the injection of capsaicin, as indicated. The distribution of the different classes of CDPs depicted in the histograms obtained during two separate recording periods of control were also highly similar to each other (see RMS values in Table 2), indicating a relatively stable pattern in the probabilities of generation of different classes of CDPs retrieved during a 20 min recording time. It can be seen as with the data obtained from experiment *300103* (Figure 7) that the CDPs with smaller amplitudes appeared more frequently than the CDPs with larger amplitudes. After the application of capsaicin, there was a substantial consistent change in the frequency of occurrence of the different classes of CDPs observed during the three recording periods. Namely, there was a clear increase in the occurrence of the largest CDPs (e.g., classes 14 and 15) and a reduction of the smallest e.g., classes 1 and 2 during the 2nd and 3rd capsaicin recordings). It should be noted that the effects of capsaicin on the probabilities of occurrence of some classes (4, 11, 14) were reduced with time. This effect has been attributed to an increased supraspinal inhibition produced by the nociceptive stimulation (Rudomin and Hernández, 2008).

**Table 2:** **RMS of the class frequencies for control and capsaicin**.

**RMS**	**ctrl_1_**	**ctrl_2_**	**capsa_1_**	**capsa_2_**	**capsa_3_**
ctrl_1_	0	0.88	5.21	5.1	3.88
ctrl_2_	0.88	0	5.31	5.22	3.99
capsa_1_	5.21	5.31	0	2.95	3.79
capsa_2_	5.1	5.22	2.95	0	2.26
capsa_3_	3.88	3.99	3.79	2.26	0

An RMS value of 0.88 between ctrl_1_ and ctrl_2_ indicates that the corresponding frequency histograms are quite similar. In contrast, the RMS between control and capsaicin histograms (capsa_1_, capsa_2_, and capsa_3_) ranged from 3.88 to 5.31, suggesting significant differences. In contrast, the RMS values between capsa_1_, capsa_2_, and capsa_3_ were below this range (2.26–3.79) suggesting more similarity, but not as significant as between control histograms.

What is most remarkable is the similarity of the histograms obtained from recordings made at different times after the injection of capsaicin (see Table 2 for RMS values between histograms). These observations demonstrate that the neuronal networks that generate spontaneous CDPs can change from a non-random dynamic state (control state) to another also non-random state after the application of capsaicin, in agreement with previous proposals based on correlation measurements (Rudomin et al., 2012; Contreras-Hernández et al., 2013).

Experiments in course are being performed to provide a more detailed characterization of the dorsal horn neurons involved in the generation of the different classes of spontaneous CDPs, particularly in view of recent observations showing that depending on the magnitude of the synchronization of the spontaneous neuronal activity within the network, the same population of dorsal horn neurons may be involved in the generation of nCDPs or npCDPs (Contreras-Hernández et al., 2015).

To validate the changes in the distribution of the different classes of CDPs selected with the present method, we examined the consistency of the dictionaries generated during the control periods as well as their variation during the capsaicin periods. In fact, we expect that a consistent good classification of classes should be obtained when comparing the classifications derived from the two control periods and that they should be somewhat different from classifications obtained from recordings made after the capsaicin injection. To this end, we estimated the significance of the deviation between pairs of histograms obtained during the five recorded periods. Namely, crtl_1_ (between 0 and 10 min), crtl_2_ (between 10 and 20 min), capsa_1_ (between 0 and 10 min post injection), capsa_2_ (between 40 and 50 min post injection) and capsa_3_ (between 60 and 70 min post injection). We assumed that such significance obeys a distribution close to the standard normal distribution if both values are taken from the same statistical population.

The observations depicted in Figure 8 demonstrate that compared with the control distributions, the intradermic injection of capsaicin increases the probabilities of generation of largest classes of CDPs. Similar variations have been observed for CDPs generated in other lumbar segments, in the same experiment as well as in five other experiments, confirming that the dictionary based methodology is able to detect ongoing non-random changes in spinal cord activity which are relatively stable during extended periods of time.

## 6. Discussion and future work

The machine learning method that we have developed is able to retrieve basic patterns of spontaneous cord dorsum potentials obtained during different experimental conditions. The present classification method comprises five phases, namely (1) CDPs candidate detection, (2) CDPs feature extraction, (3) CDPs cluster analysis, (4) CDPs occurrence analysis, and (5) CDPs recurrence analysis (see Section 2.5). The first three phases are addressed by the present paper while we have left the last two for future work.

The main advantages of this method are:

- the automation of the process of selecting CDPs candidates from raw recordings when compared with the manual selection;
- it is not constrained to search for known CDPs but allows to discover (and in fact does so) new classes of CDPs (see Section 5.1),
- it performs an exhaustive exploration of the whole set of data,
- it reduces the time of analysis in several orders of magnitude from days to tens of minutes.

Another relevant characteristic is its the capability of identifying a basic dictionary of CDPs classes that show statistical stability through different experimental manipulations. These dictionaries are also able to confirm (and to extend) the expert knowledge, characterizing changes in the CDPs activity and identifying patterns in raw data recorded under normal and pathological situations that would remain undetected with the previously used selection methods. In the third place, the dictionaries are able to detect changes in the state of the system, facilitating the analysis of the effects of different manipulations of the experiment.

As a drawback, the labeling of the CDPs classes can not be fully automated and requires some expert's analysis. The expert has to examine the different classes of spontaneous CDPs and decide which of them may be of functional relevance based on the information provided by a detailed characterization of the functional features of the neurons involved in their generation, as it has been done with the spontaneous nCDPs and npCDPs (Chávez et al., 2012; Contreras-Hernández et al., 2015).

From a physiological point of view, fractal analysis of the spontaneous CDPs (Rodríguez et al., 2010) recorded from the lumbosacral segments has shown that these potentials are not random but have an underlying temporal and spatial structure. This leads to the question on the extent to which spontaneous CDPs with the same shape and amplitude are recurrently generated by the activation of relatively stable, highly coherent neuronal networks compatible with the concept of *modular* organization, an issue that has been used to explain the muscular synergies during the execution of a variety of motor behaviors (Bizzi et al., 2008), still a matter of controversies (Rudomin, 2009).

The present results provide some insight on this question by showing that under control conditions there is a repertoire of different classes of spontaneous CDPs, which appear with a highly stable frequency in different periods during the control conditions (Figure 8). After the application of capsaicin the frequency of generation of some classes of the spontaneous CDPs changes, again with a relatively stable profile along different periods (see Table 2). It is in agreement with previous observations where it was demonstrated that the profile of the correlation between continuous records from different pairs of lumbar segments remains highly stable during control conditions and changes by the intradermic injection of capsaicin (Rudomin et al., 2012; Contreras-Hernández et al., 2013). Quite interestingly, the changes in frequency of the CDPs from the control condition to the capsaicin condition appear to be more marked for the CDPs recorded in the left side of the spinal cord, ipsilaterally to the site of application of the capsaicin as indicated by the RMS coefficients. Besides the implications of these findings on hyperalgesia and pain perception, that will be dealt in a forthcoming publication, the question remains on the extent to which each class of CDPs is generated by the synchronous activity of particular sets of dorsal horn neurons or if they are produced by a distributed set of dorsal horn neurons that may acquire different configurations of internal connectivity, as it seems to be the case with the nCDPs and npCDPs.

Finally, the present approach for classification of spontaneous neuronal population potentials based on their shapes and amplitudes has possible clinical applications, for example in the analysis of spontaneous spinal cord potentials in patients with peripheral nerve, root and spinal cord disorders (Liang et al., 2015), of changes in EEG during different levels of anesthesia in human patients (Ertekin et al., 1983), or in the control of electrocorticographic based neuroprosthesis (Chao et al., 2001; Anderson et al., 2012).

### Conflict of interest statement

The authors declare that the research was conducted in the absence of any commercial or financial relationships that could be construed as a potential conflict of interest.

## A. Appendix

### A.1. measure for comparing histograms

Let us consider a simple model with two histograms where the random variable in each bin obeys the normal distribution

(A1) $$\begin{array}{r} p\left(x|n_{ik}\right)=\frac{1}{\sqrt{2\pi \sigma_{ik}}}e^{-\frac{{\left(x-n_{ik}\right)}^{2}}{2\sigma_{ik}}} \end{array}$$

where the expected value in the bin *i* is equal to *n*_*ik*_ and the variance $\sigma_{ik}^{2}=n_{ik}$ and *k* is the histogram number (*k* = 1, 2). We define the significance as

(A2) $$\begin{array}{r} {Ŝ}_{i}=\frac{{\hat{n}}_{i1}-{\hat{n}}_{i2}}{\sqrt{{\hat{\sigma }}_{i1}^{2}+{\hat{\sigma }}_{i2}^{2}}} \end{array}$$

where ${\hat{n}}_{ik}$ is an observed value in the bin *i* of the histogram *k* and ${\hat{\sigma }}_{ik}^{2}={\hat{n}}_{ik}$. This model can be considered as the approximation of the Poisson distribution by the Normal distribution. The values *n*_*ik*_, (*i* = 1, 2, …, *M, k* = 1, 2) are the numbers of events appeared in the bin *i* for the histogram *k*. We consider the *RMS* (the root mean square) of the distribution of the significances

(A3) $$RMS=\sqrt{\frac{\sum_{i=1}^{M}{\left({\hat{S}}_{i}-\bar{S}\right)}^{2}}{M}}$$

Here $\bar{S}$ is the mean value of Ŝ_*i*_. The *RMS* measures the distance between two histograms. If total number of events *N*_1_ in the histogram 1 and total number of events *N*_2_ in the histogram 2 are different, then the normalized significance Ŝ_*i*_(*K*) is calculated as follows

(A4) $$\begin{array}{r} {Ŝ}_{i}\left(K\right)=\frac{{\hat{n}}_{i1}-K{\hat{n}}_{i2}}{\sqrt{{\hat{\sigma }}_{i1}^{2}+K^{2}{\hat{\sigma }}_{i2}^{2}}} \end{array}$$

where *K* = *N*_1_ / *N*_2_. The relation $RMS^{2}=\chi^{2}∕M-{\bar{S}}^{2}$ exists for the distribution of significances where $\chi^{2}=\overset{M}{\underset{i=1}{\sum }}{Ŝ}_{i}^{2}$. One can show that the distribution of observed significances is close to normal distribution with the *RMS*~1. This distance measure between two histograms has a clear interpretation: *RMS*~0 histograms are identical, *RMS*~1 both histograms are obtained from the same parent distribution, *RMS* ≫ 1 histograms are obtained from different parent distributions.
